# Sex-specific extracerebral complications in patients with aneurysmal subarachnoid hemorrhage

**DOI:** 10.3389/fneur.2023.1098300

**Published:** 2023-05-10

**Authors:** Stefan Y. Bögli, Sabrina Beham, Laura Hirsbrunner, Friederike Nellessen, Francesca Casagrande, Emanuela Keller, Giovanna Brandi

**Affiliations:** ^1^Neurocritical Care Unit, Department of Neurosurgery, Institute for Intensive Care Medicine, University Hospital Zurich, Zurich, Switzerland; ^2^Department of Neurology, University Hospital Zurich, Zurich, Switzerland; ^3^Clinical Neuroscience Center, University Hospital Zurich and University of Zurich, Zurich, Switzerland

**Keywords:** subarachnoid hemorrhage, extracranial complications, gender medicine, outcome, delayed cerebral ischemia, stroke

## Abstract

**Background:**

Extracerebral complications in patients with aneurysmal subarachnoid hemorrhage (aSAH) often occur during their stay at the neurocritical care unit (NCCU). Their influence on outcomes is poorly studied. The identification of sex-specific extracerebral complications in patients with aSAH and their impact on outcomes might aid more personalized monitoring and therapy strategies, aiming to improve outcomes.

**Methods:**

Consecutive patients with aSAH admitted to the NCCU over a 6-year period were evaluated for the occurrence of extracerebral complications (according to prespecified criteria). Outcomes were assessed with the Glasgow Outcome Scale Extended (GOSE) at 3 months and dichotomized as favorable (GOSE 5–8) and unfavorable (GOSE 1–4). Sex-specific extracerebral complications and their impact on outcomes were investigated. Based on the results of the univariate analysis, a multivariate analysis with unfavorable outcomes or the occurrence of certain complications as dependent variables was performed.

**Results:**

Overall, 343 patients were included. Most of them were women (63.6%), and they were older than men. Demographics, presence of comorbidities, radiological findings, severity of bleeding, and aneurysm-securing strategies were compared among the sexes. More women than men suffered from cardiac complications (*p* = 0.013) and infection (*p* = 0.048). Patients with unfavorable outcomes were more likely to suffer from cardiac (*p* < 0.001), respiratory (*p* < 0.001), hepatic/gastrointestinal (*p* = 0.023), and hematological (*p* = 0.021) complications. In the multivariable analysis, known factors including age, female sex, increasing number of comorbidities, increasing World Federation of Neurosurgical Societies (WFNS), and Fisher grading were expectedly associated with unfavorable outcomes. When adding complications to these models, these factors remained significant. However, when considering the complications, only pulmonary and cardiac complications remained independently associated with unfavorable outcomes.

**Conclusion:**

Extracerebral complications after aSAH are frequent. Cardiac and pulmonary complications are independent predictors of unfavorable outcomes. Sex-specific extracerebral complications in patients with aSAH exist. Women suffered more frequently from cardiac and infectious complications potentially explaining the worse outcomes.

## Introduction

In patients with aneurysmal subarachnoid hemorrhage (aSAH), the high mortality and morbidity are not only associated with the initial bleeding but also with intracerebral secondary complications, such as rebleeding, delayed cerebral ischemia (DCI), and hydrocephalus (1). Similar to intracerebral complications, extracerebral complications are frequent (with reviews finding cardiac injury in ~36%, arrhythmias in ~35%, and acute respiratory distress syndrome in ~4–18% of patients covering multiple studies) (2). Cardiovascular and pulmonary complications are the most frequent and are associated with a sudden and sustained increase in systemic catecholamines, which can lead to organ dysfunction, hypoxemia, hyperglycemia, and an inflammatory state with the release of cytokines (3). In particular, cardiac complications and markers of cardiac injury have been shown to be associated with unfavorable outcomes and the occurrence of DCI (4) with other studies proving the benefit of combined multi-organ dysfunction evaluation for the prediction of outcomes (5). Fever, anemia, and hyperglycemia have also been found to be associated with increased mortality and poor outcomes (6).

Sex-specific differences exist in almost every aspect of a disease. Women are more likely to be affected by aSAH (7–10) possibly due to the intrinsic weakness of the vessel walls, collagen, and elastin interference factors, as well as hormonal aspects (11, 12). Furthermore, aneurysm location itself also appears to be associated with sex (13, 14), with more aneurysms located along the internal carotid artery in female subjects and along the anterior cerebral artery in male subjects. However, so far, while complications after aSAH have been elucidated in various studies, the presence of sex-specific extracerebral complications during the stay at the neurocritical care unit (NCCU) is less investigated.

In this retrospective study, we focus on the extracerebral consequences of aSAH, particularly on sex-specific complications during the stay at the NCCU and their influence on outcomes. The identification of these might aid personalized management and treatment strategies.

## Materials and methods

We retrospectively reviewed the medical records of all consecutive patients with aSAH admitted to the NCCU of the University Hospital Zurich over a 6-year period (January 2016–December 2021). All adults (≥18 years old) admitted with aSAH (i.e., with imaging evidence of a ruptured aneurysm) were eligible for inclusion. Exclusion criteria were as follows: (1) patients with only unruptured, traumatic, fusiform, dissecting, or mycotic aneurysms and (2) patients' written or documented oral refusal to have their data analyzed for research projects. The study was performed in accordance with the ethical standards laid down in the 2013 Declaration of Helsinki. The local ethics committee approved the study. STROBE guidelines were used to draft the manuscript.

### Patients' population and management

Patients were treated according to the latest AHA/ASA guidelines (15). These include the following: (1) early aneurysm securing by surgical or endovascular means; (2) external ventricular drain insertion (EVD) (BACTISEAL^®^ EVD Catheter, CODMAN, Johnson & Johnson, Raynham, MA, USA) in case of enlargement of the third ventricle and the temporal horns of the lateral ventricles (i.e., ventriculomegaly with signs of acute occlusive hydrocephalus) for the monitoring of intracerebral pressure (ICP) in unconscious and comatose patients; and (3) insertion of invasive multimodal neuromonitoring, including cerebral microdialysis (CMA 70, CMA Microdialysis, Solna, Sweden), brain tissue oxygenation monitoring (LiCox system, Integra Neurosciences, Plainsboro, NJ), and continuous electroencephalography in case of prolonged impaired consciousness either due to the severity of the disease itself or due to the need for deep sedation during the vasospasm phase.

At least until day 14 after aSAH—during the vasospasm phase, all patients (irrespective of initial clinical or radiographic grade) are monitored and treated at the NCCU. In the case of symptomatic vasospasm confirmed by CT-angiography with corresponding clinical deterioration or corresponding perfusion deficit upon perfusion imaging, controlled arterial hypertension is induced by the administration of norepinephrine. In case of non-response to hemodynamic therapy, intraarterial spasmolysis—available 24 h per day—is performed.

### Data collection

Data collection was performed by scanning the electronic health records (KISIM-TM; Cistec^®^ Zurich, Switzerland) for demographic characteristics, and clinical course during the ICU stay.

Demographic data collected were sex, age, and presence of comorbidities, based on the Charlson Comorbidity Index (CCI) (16) (i.e., history of myocardial infarction, congestive heart failure, peripheral vascular disease, history of the cerebrovascular event, dementia, chronic pulmonary disease, rheumatologic disease, gastric ulcer, liver disease, diabetes with or without chronic complications, kidney disease, and history of cancer). The severity of bleeding was assessed by the WFNS and Fisher scale. Complications during the stay at the NCCU were collected in an organ system-specific manner (Supplementary material 1) as follows:

– Cardiovascular (including acute coronary syndrome, Takotsubo syndrome, arrhythmic disorders, and other cardiovascular disturbances);
– Pulmonary (including acute respiratory distress syndrome, aspiration pneumonia, ventilator-associated pneumonia, hospital-acquired pneumonia, and chronic obstructive pulmonary reactivation);
– Hepatic and gastrointestinal (paralytic ileus, peptic ulcer, abdominal compartment syndrome, mesenteric ischemia, acute on chronic liver failure, transaminitis, acute pancreatitis, and cholestatic injury);
– Renal (including acute kidney injury);
– Infections (including urogenital infections, catheter-related bloodstream infection, and sepsis/septic shock);
– Electrolyte disturbances (including hypokalemia, hyperkaliemia, hypomagnesemia, hypermagnesemia, hypophosphatemia, and hyperphosphatemia; sodium disorders are excluded);
– Sodium disorders (including diabetes insipidus, cerebral salt wasting syndrome, and syndrome of inappropriate antidiuretic hormone secretion);
– Hematologic (including hemorrhagic shock and active bleeding but not in the cerebral nervous system);
– Thromboembolic events (including deep vein thrombosis, intravascular catheter-related thrombosis, and pulmonary embolism).

The outcome is reported using the Glasgow Outcome Scale Extended (GOSE) extracted from routine follow-up consultations at 3 months (which include a neurological examination, as well as a description of current occupation including the percentage of working capability). A dichotomized GOSE in favorable (GOSE 5–8) and unfavorable (GOSE 1–4) was considered in the analysis, as in previous studies (10, 17). After excluding infarction caused by aneurysm securing, DCI was defined as a cerebral infarction on CT scans or magnetic resonance images, combined with either clinical deterioration (new focal neurological deficit or GCS decrease of 2 points) and/or impaired perfusion in CT- or MR perfusion (18, 19).

### Statistical analysis

Statistical analysis was performed using SPSS version 26. Data were dichotomized by sex (male vs. female) or outcome (favorable vs. unfavorable). Descriptive statistics are reported as counts/percentages, mean ± standard deviation (SD), or as median including the interquartile range (IQR) as appropriate. All continuous data were tested for normality using Shapiro–Wilk's test. Univariate logistic regression was used to find variables associated with sex and unfavorable outcomes. Multivariate analysis was performed based on the univariate analysis to correct results for the differences in clinical characteristics with unfavorable outcomes as a dependent variable. Respective odds ratios (OR) including 95% confidence intervals (95%-CI) are only shown for significant associations. The significance level was set at a *p*-value of < 0.05.

## Results

Overall, 343 patients fulfilled the inclusion criteria. Of these, 63.6% were women (*N* = 218). Women were older than men (*p* = 0.002, an average of 5 years). In addition, no differences by sex were found considering the presence of comorbidities, the severity of bleeding, radiographic findings on the first CT scan (presence of intracerebral hemorrhage, ventriculomegaly, and intraventricular hemorrhage), and treatment modality (coiling/ clipping), as shown in Table 1.

**Table 1:** Univariate analysis: demographics/characteristics^*^.

		**Male**	**Female**	***p*** **-value**	**OR**	**95% CI**
		125 (36.4)	218 (63.6)			
Age (mean ± SD)		54.38 ± 11.6	59.15 ± 13.8	< 0.01	1.028	1.011–1.046
Length of stay ICU [median (IQR)]		14 [12, 22]	15 [10, 22]	0.58		
CCI [median (IQR)]		0 [0, 2]	0 [0, 1.25]	0.71		
WFNS	1	38 (30.4)	78 (35.8)	0.44		
	2	28 (22.4)	43 (19.7)			
	3	3 (2.4)	10 (4.6)			
	4	28 (22.4)	40 (18.3)			
	5	28 (22.4)	47 (21.6)			
GOSE at 3 months	1	17 (13.8)	43 (20.0)	0.18		
	2	3 (2.4)	9 (4.2)			
	3	21 (17.1)	32 (14.9)			
	4	7 (5.7)	26 (12.1)			
	5	22 (17.9)	23 (10.7)			
	6	22 (17.9)	17 (7.9)			
	7	13 (10.6)	41 (19.1)			
	8	18 (14.6)	24 (11.2)			
Aneurysm location (anterior)		102 (81.6)	169 (77.5)	0.37		
Fisher	1	4 (3.2)	9 (4.1)	0.99		
	2	8 (6.4)	16 (7.4)			
	3	56 (44.8)	87 (40.1)			
	4	57 (45.6)	105 (48.4)			
ICH		37 (29.6)	58 (26.6)	0.55		
Ventriculomegaly		65 (52.0)	114 (52.3)	0.95		
IVH		87 (69.0)	154 (70.6)	0.78		
Treatment modality (clipping)		60 (48.0)	99 (45.4)	0.31		
DCI		39 (31.2)	59 (27.1)	0.41		

* Univariate logistic regression evaluating the association of demographics and clinical features to sex. Data shown as number (%) unless otherwise stated. SD, standard deviation; IQR, inter-quartile range; OR, odds ratio; CI, confidence interval; ICU, intensive care unit; CCI, Charlson Comorbidity Index; WFNS, World Federation of Neurosurgeons Society; GOSE, Glasgow Outcome Scale Extended; ICH, intracerebral hemorrhage; IVH, intraventricular hemorrhage; DCI, delayed cerebral ischemia.

The extracerebral complications are listed in Table 2. More women than men suffered from cardiac complications (*p* = 0.013), particularly arrhythmic disorders (*p* = 0.019). Infectious diseases, overall, were more frequent in women than men (*p* = 0.048). More women suffered from urogenital tract infections (*p* = 0.002). Pulmonary complications were frequent, but no sex-related differences were found. Female sex also remained an independent predictor of cardiac complications after correction for age (*p* = 0.037, OR 1.837, 95% CI 1.036–3.257) but not for infectious complications when corrected for age (*p* = 0.054).

**Table 2:** Univariate analysis: complications^*^.

	**Male**	**Female**	***p*** **-value**	**OR**	**95% CI**
Any	92 (73.6)	174 (79.8)	0.19		
Cardiac	20 (16.0)	61 (28.0)	0.01	2.040	1.163–3.579
Acute coronary syndrome	9 (7.2)	11 (5.1)	0.41		
arrhytmogenic	11 (8.8)	40 (18.4)	0.02	2.329	1.148–4.725
Tako-Tsubo	2 (1.6)	14 (6.4)	0.06		
Pulmonary	64 (51.2)	93 (42.7)	0.13		
Infectious	20 (16.0)	55 (25.2)	0.04	1.771	1.004–3.125
Urinary tract infection	6 (4.8)	37 (17.0)	< 0.01	4.054	1.660–9.903
Other	8 (6.4)	9 (4.1)	0.36		
Sepsis	7 (5.6)	14 (6.4)	0.76		
Hepatic/gastrointestinal	5 (4.0)	15 (6.9)	0.28		
Renal	8 (6.4)	12 (5.5)	0.73		
Acute-on-chronic renal failure	3 (2.4)	5 (2.3)	0.95		
Acute renal failure	2 (1.6)	4 (1.8)	0.87		
Rhabdomyolysis	2 (1.6)	1 (0.5)	0.31		
Polyuria	0 (0.0)	2 (0.9)	0.99		
Electrolytes	39 (31.2)	72 (33.0)	0.73		
Sodium	36 (28.8)	70 (32.1)	0.52		
Other	3 (2.4)	3 (1.4)	0.49		
Hematologic	16 (12.8)	30 (13.8)	0.80		
Extracerebral hemorrhage	7 (5.6)	10 (4.6)	0.68		
Thrombosis	5 (4.0)	14 (6.4)	0.35		
Thrombocytopenia	3 (2.4)	8 (3.7)	0.52		
Other coagulopathy	3 (2.4)	2 (0.9)	0.29		

* Univariate logistic regression evaluating the association of complications to sex. Data are shown as numbers (%). OR, odds ratio; CI, confidence interval; SD, standard deviation; IQR, inter-quartile range.

Considering outcomes, female sex, age, CCI, WFNS, and Fisher grading were associated with unfavorable outcomes in the univariate analysis. Considering the extracerebral complications, cardiac, pulmonary, hepatic/gastrointestinal, and hematologic complications were associated with unfavorable outcomes. In the multivariate analysis, increasing age, WFNS, and Fisher grading remained independent predictors of unfavorable outcomes irrespective of the addition of extracerebral complications. When these were added, aside from the above-mentioned known predictors, only pulmonary and cardiac complications remained independent predictors of unfavorable outcomes (Table 3). Sex was an independent predictor of unfavorable outcomes when considered univariately in the base model (excluding extracerebral complications) as well as in the multivariable analysis when adding pulmonary, hepatic/gastrointestinal, or hematologic complications. Interestingly, however, the female sex was not an independent predictor of unfavorable outcomes when including cardiac complications in the base model (Table 4) possibly due to the high association between sex and cardiac complications (*p* = 0.01, OR 2.040, 95% CI 1.163–3.579).

**Table 3:** Univariate prediction: unfavorable outcome^*^.

**Demographics**	***p*** **-value**	**OR**	**95% CI**
Age	<0.01	1.047	1.029–1.066
Sex (female)	0.02	1.698	1.078–2.674
LOS_ICU	0.09		
CCI	<0.01	1.357	1.147–1.606
WFNS	<0.01	1.866	1.595–2.183
Aneurysm location (anterior)	0.68		
Fisher	<0.01	4.541	2.999–6.878
ICB	<0.01	3.637	2.175–6.083
Ventriculomegaly	<0.01	3.857	2.444–6.088
IVH	<0.01	5.208	3.011–9.006
Treatment modality (clipping)	0.97		
DCI	0.02	1.783	1.100–2.888
**Complications**			
Cardiac	<0.01	4.206	2.380–7.433
Pulmonary	<0.01	6.612	4.092–10.686
Infectious	0.49		
Hepatic/gastrointestinal	0.02	3.348	1.178–9.520
Renal	0.46		
Electrolytes	0.30		
Hematological	0.02	2.171	1.126–4.185

* Univariate logistic regression for the prediction of unfavorable outcomes. SD, standard deviation; IQR, inter-quartile range; OR, odds ratio; CI, confidence interval; LOS ICU, length of stay intensive care unit; CCI, Charlson Comorbidity Index; WFNS, World Federation of Neurosurgeons Society; GOSE, Glasgow Outcome Scale Extended; ICH, intracerebral hemorrhage; IVH, intraventricular hemorrhage; DCI, delayed cerebral ischemia.

**Table 4:** Multivariate prediction without (base model) and with complications: unfavorable outcomes^*^.

**Basemodel**	***p*** **-value**	**OR**	**95% CI**
Age (per year increase)	<0.01	1.037	1.014–1.060
Sex (female)	0.02	1.943	1.097–3.442
CCI (per point increase)	0.03	1.235	1.019–1.497
WFNS (per step increase)	<0.01	1.633	1.360–1.960
Fisher (per step increase)	<0.01	2.995	1.871–4.794
**Basemodel** + **cardiac**			
Age (per year)	<0.01	1.036	1.014–1.060
Sex (female)	0.06	1.748	0.978–3.123
CCI (per point increase)	0.07	1.201	0.987–1.463
WFNS (per step increase)	<0.01	1.555	1.289–1.877
Fisher (per step increase)	<0.01	3.052	1.886–4.939
Cardiac complications	0.02	2.196	1.108–4.352
**Basemodel** + **pulmonary**			
Age (per year)	<0.01	1.035	1.011–1.059
Sex (female)	<0.01	2.594	1.389–4.847
CCI (per point increase)	0.05	1.22	0.998–1.492
WFNS (per step increase)	<0.01	1.508	1.243–1.830
Fisher (per step increase)	<0.01	2.72	1.668–4.434
Pulmonary complications	<0.01	4.719	2.625–8.484
**Basemodel** + **hepatic/gastrointestinal**			
Age (per year)	<0.01	1.038	1.015–1.061
Sex (female)	0.03	1.885	1.060–3.350
CCI (per point increase)	0.03	1.237	1.022–1.499
WFNS (per step increase)	<0.01	1.609	1.338–1.933
Fisher (per step increase)	<0.01	3.030	1.889–4.859
Hepatic/gastrointestinal	0.24	2.100	0.610–7.239
**Basemodel** + **hematologic**			
Age (per year)	0.01	1.039	1.016–1.062
Sex (female)	0.03	1.931	1.087–3.432
CCI (per point increase)	0.04	1.223	1.008–1.484
WFNS (per step increase)	<0.01	1.619	1.348–1.944
Fisher (per step increase)	<0.01	3.077	1.906–4.937
Hematologic	0.11	1.899	0.866–4.165

* Multivariate logistic regression for the prediction of unfavorable outcomes either using known predictors (Basemodel) or including systemic complications. OR, odds ratio; CI, confidence interval; LOS ICU, length of stay intensive care unit; CCI, Charlson Comorbidity Index; WFNS, World Federation of Neurosurgeons Society.

## Discussion

In this study, we investigated the frequency of extracerebral complications in patients suffering from aSAH with a particular focus on sex-related differences and their influence on outcomes. Extracerebral complications in patients with aSAH are frequent. Wartenberg et al. described at least one medical complication in 79% of patients with aSAH (6). Similarly, Solenski et al. reported that all the patients recruited had one or more medical complications (20). We found that both sexes frequently suffered from complications (female: 79.8%, male 73.6%). We found cardiac and pulmonary complications to be the most frequent complications in this study population. Similar to prior reports, they were independent predictors of unfavorable outcomes (21–24). No specific subtype of pulmonary complication was associated with significantly worse outcomes, confirming prior reports (22). A catecholamine storm following acute brain injury has been proposed to lead to extracerebral complications by means of hypoxemia, hyperglycemia, and inflammatory state due to the release of pro-inflammatory cytokines (25). A similar mechanism could also explain the complications found in aSAH.

**Figure 1:**
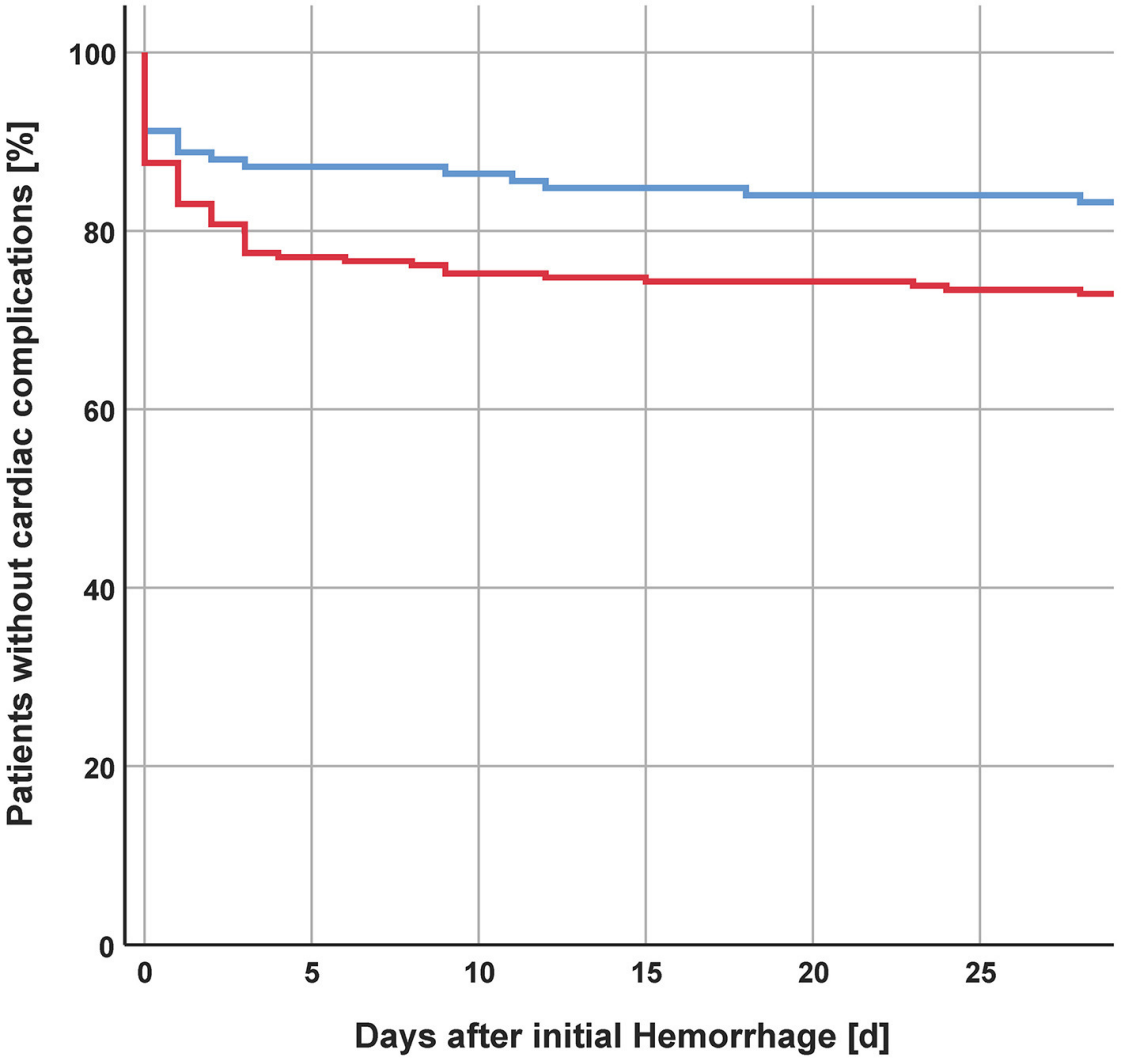
Incidence of cardiac complications after initial hemorrhage.

Cardiac complications and the combination of multi-organ dysfunction after aSAH have been shown to be associated with unfavorable outcomes (4, 5). However, whether extracerebral complications occur in a sex-specific manner remains poorly investigated. In our cohort, women suffered more frequently from cardiac complications even after correction for their older age. This difference was mostly based on the higher frequency of arrhytmogenic complications in female subjects. Interestingly, both the absolute frequency, as well as the lack of sex-related difference in the frequency of Takotsubo syndrome, differs from current reviews most likely due to the need for a transthoracic echocardiogram for its diagnosis, which is only ordered in patients with clinical suspicion (26). As previously described, women presented a worse outcome after aSAH (10). Possibly the difference in cardiac complication frequency might explain this difference. In our cohort, cardiac complications occurred mostly in women and mostly within the first 4 days after the initial hemorrhage (Figure 1). aSAH leads to an acute increased sympathetic nervous system activity with an increased release of catecholamines (27). This increased activity is also associated with cardiac complications and in particular Takotsubo syndrome (26). Some studies even report the beneficial effects of beta-blockers in patients with aSAH (28, 29). Standardized use of beta-blockers in aSAH, however, has not been established yet. Patients at risk for symptomatic vasospasm and DCI receive controlled arterial hypertension to improve cerebral perfusion. Induced hypertension may lead to serious adverse events, such as cardiac arrhythmia, myocardial infarction, pulmonary edema, brain edema, hemorrhagic infarction, and rebleeding (30). These adverse effects of induced hypertension might be potentiated in patients who already suffered cardiac complications/injury prior to its induction.

In the study population, women were more likely to suffer from infections, particularly urogenital infections, during their stay at the NCCU. This finding is in line with previous reports (31, 32). Anatomical differences and the use of bladder catheters might increase the risk of infections in women. This finding suggests that the use of bladder catheters should be carefully evaluated, and removal—in female patients, should be reconsidered on a daily basis in order to prevent the development of urogenital infections.

### Strengths and limitations

Our study has several strengths. First, we collected data on a large number of extracerebral complications. Second, we decided to focus on the currently poorly described presence of sex-specific extracerebral complications. Our findings are of clinical relevance and might help to improve the outcomes of patients with aSAH. There are also limitations to our study. First, this is a retrospective single-center study, limiting its generalizability. Second, despite the large number of extracerebral complications collected, some of the interests might not have been investigated, permitting only speculations on the reported differences by sex. Third, we limited our analysis to the duration of stay at the NCCU (at least the first 14 days after the initial bleeding) with no information on complications that developed later on.

## Conclusion

Extracerebral complications during the stay at the NCCU after aSAH are frequent. Cardiac and pulmonary complications are predictors of unfavorable outcomes. There are sex-specific extracerebral complications. Women more commonly suffer from cardiac and infectious complications. Patients with prior cardiac injury might benefit from personalized management when at risk of symptomatic vasospasm/DCI with either closer evaluation for further cardiac injury or possibly lower blood pressure target values. Due to the increased risk of urogenital infections in women, the use of bladder catheters should be carefully evaluated, and early removal should be advised.

## Data availability statement

The raw data supporting the conclusions of this article will be made available by the authors, without undue reservation.

## Ethics statement

The studies involving human participants were reviewed and approved by Kantonale Ethik-Kommission Zürich. The patients/participants provided their written informed consent to participate in this study.

## Author contributions

SBö: conceptualization, statistical analysis, critical revision, and data interpretation. SBe: data acquisition, writing—original draft, and data interpretation. LH: writing—original draft and data acquisition. FN and FC: critical revision and data acquisition. GB: supervision, writing—review and editing, project administration, and conceptualization. EK: supervision, writing—review and editing, and data interpretation. All authors contributed to the article and approved the submitted version.
