# Supplementary material for: Sex-specific extracerebral complications in patients with aneurysmal subarachnoid hemorrhage
Source: Front Neurol. 2023 May 10;14:1098300. doi: 10.3389/fneur.2023.1098300 (PMC10206055; doi:10.3389/fneur.2023.1098300)
Supplement: Supplementary file 1 [file Table_1.docx]

| **Organ System** | **Complication** | **Definition** |
| --- | --- | --- |
| **Cardiovascular** | Acute coronary syndrome | Rise and / or fall of troponin values with at least 1 value above the 99th percentile upper reference limit and with at least 1 of the following:   - Clinical symptoms of myocardial ischemia - New ischemic ECG-changes - Development of pathological Q-waves - New regional wall motion abnormality in a pattern consistent with ischemic etiology   Identification of a coronary thrombus by angiography including intracoronary imaging or by autopsy [1] |
|  | Takotsubo Syndrome | - Transient regional wall motion abnormalities of LV or RV myocardium, usually extending beyond a single epicardial vascular distribution and often result in circumferential dysfunction of the ventricular segments involved (evaluated by a cardiologist) - Absence of culprit atherosclerotic coronary artery disease or other pathological conditions like hypertrophic cardiomyopathy, viral myocarditis - New and reversible ECG abnormalities [2] |
|  | Arrhythmic disorders | All arrhythmias needing intervention (hemodynamic instability leading to increased dosing of catecholamines, application of antiarrhythmic drugs or electric cardioversion),   - detected through PDMS or documented in ICU discharge papers. |
| **Pulmonary** | ARDS | - According to the Berlin Definition 2012 [3] |
|  | Aspiration pneumonia | Fever, sputum or impaired oxygenation, inflammatory findings on chest radiography and overt / strongly suspected aspiration |
|  | Ventilator-associated pneumonia | Hospital-acquired pneumonia developing after > 48 hours of ventilation [4] |
|  | Hospital-acquired pneumonia | Pneumonia developing > 48 hours after hospital admission [4] |
|  | Chronic obstructive pulmonary disease exacerbation | Worsening of COPD symptoms, as more coughing, wheezing, or shortness of breath than usual; changes in color, thickness, or amount of mucus. |
| **Hepatic and Gastrointestinal** | Paralytic Ileus | Functional blockage of the small and/or large intestine resulting in elevated gastric residual volume (> 500ml in 6 hours) and administration of procinetic drugs (i.e. Metoclopramide, Erythromycin, Dexpanthenol or Neostigmine) [5] |
|  | Peptic ulcer | Upper gastrointestinal mucosal lesions found endoscopically [6] |
|  | Abdominal compartment syndrome | Sustained IAP > 20mmHg that is associated with new organ dysfunction / failure [7] |
|  | Mesenteric ischemia | Sudden interruption or reduction of the blood supply to a segment of the small intestine, resulting in an insufficiency to meet the metabolic demands of the visceral organs [8], diagnosed by surgical inspection or through imaging |
|  | Acute or acute on chronic liver failure | Severe liver injury, potentially reversible in nature and with onset of hepatic encephalopathy within 8 to 28 days of the first symptoms in the absence of pre-existing liver disease [9], clinically manifesting in hepatic dysfunction, abnormal liver biochemical values, and coagulopathy |
|  | Transaminitis | Alanin-aminotransferase ≥ 2x URL |
|  | Acute pancreatitis | Two of the following three features:   - abdominal pain consistent with acute pancreatitis - serum lipase activity (or amylase activity) at least three times greater than the URL   characteristic findings of acute pancreatitis on contrast-enhanced CT, less commonly MRI or transabdominal ultrasonography [10] |
|  | Cholestatic injury | Alkaline phosphatase ≥1.5 x URL and gamma-glutamyl transferase ≥ 3x URL [11] |
| **Renal** | Acute kidney injury | Elevation of creatinine ≥1.5x over baseline or ≥ 26.5mmol/L increase in creatinine for 6-12h [12] |
| **Infectious** | Urogenital infection | Positive cultures obtained from urine, urethral sample, vaginal sample |
|  | Catheter-related bloodstream infection | Bacteriaemia originating from an intravenous catheter: positive blood cultures drawn from the catheter and proof of bacterial growth on said catheter after removal [13] |
|  | Sepsis/ Septic shock | Sepsis plus vasopressor requirement to maintain a mean arterial pressure of 65 mm Hg or greater and serum lactate level greater than 2 mmol/L in the absence of hypovolemia [14] |
| **Electrolytes disturbances** | Hypokalemia | Serum kalium <3.5 mmol/l |
|  | Hyperkalimia | Serum kalium >5.5 mmol/l |
|  | Hypomagnesemia | Serum magnesium <0.66 mmol/l |
|  | Hypermagnesemia | Serum magnesium >1.07 mmol/l |
|  | Hypophosphatemia | Serum phosphate <0.87 mmol/l |
|  | Hyperphosphatemia | Serum phosphate >1.45 mmol/l |
| **Sodium disorders** | Diabetes insipidus | Polyuria (> 3L/24h) and urine osmolality <300 mOsm/kg H2O, leading to hypernatraemia [15] |
|  | Cerebral salt wasting syndrome | Hyponatraemia with increased urinary sodium concentration and hypovolemia in the setting of acute intracranial disease [16] |
|  | Syndrome of inadequate ADH secretion | Diagnosis of exclusion, hyponatraemia associated with a serum hypoosmolality of less than 275 mOsm/kg H2O, urine osmolality > 100mOsm/kgH2O, urine sodium > 30mmol/L [17] |
| **Hematological** | Hemorrhagic shock | Massive transfusion (4 PRBCs in 1 hour and continuous need for transfusion) vasopressor support and signs of organ hypoperfusion (lactate ≥ 2mmol/L, oliguria ≤0.5ml/kg/h) [18] |
|  | Active bleeding | Any bleeding from a site other than intracerebral requiring PBRC administration |
| **Thromboembolic** | Deep vein thrombosis | Occlusion of a deep vein, diagnosed by compression ultrasonography or angio-CT-scan |
|  | Catheter-related thrombosis | Thrombosis associated with a central venous line, diagnosed through angio-CT-scan or Doppler-ultrasound |
|  | Pulmonary embolism | Embolism in the pulmonary vasculature, detected through CT pulmonary angiography |

Abbreviations:

ADH, antidiuretic hormone. ARDS, acute respiratory distress syndrome. CSF, cerebrospinal fluid. CT, computed tomography. ECG, electrocardiography. IAP, intra-abdominal pressure. ICU, intensive care unit. LV, left ventricle. LVEF, left-ventricular ejection fraction. MAP, mean arterial pressure. MRI, magnetic resonance imaging. PBRC, packed red blood cells. PDMS, patient data management system. SBP, systolic blood pressure. URL, upper reference limit.

1. Thygesen, K., et al., *Fourth Universal Definition of Myocardial Infarction (2018).* Circulation, 2018. **138**(20): p. e618-e651.

2. Lyon, A.R., et al., *Current state of knowledge on Takotsubo syndrome: a Position Statement from the Taskforce on Takotsubo Syndrome of the Heart Failure Association of the European Society of Cardiology.* Eur J Heart Fail, 2016. **18**(1): p. 8-27.

3. Force, A.D.T., et al., *Acute respiratory distress syndrome: the Berlin Definition.* JAMA, 2012. **307**(23): p. 2526-33.

4. American Thoracic, S. and A. Infectious Diseases Society of, *Guidelines for the management of adults with hospital-acquired, ventilator-associated, and healthcare-associated pneumonia.* Am J Respir Crit Care Med, 2005. **171**(4): p. 388-416.

5. Blaser, A.R., et al., *Definition, prevalence, and outcome of feeding intolerance in intensive care: a systematic review and meta-analysis.* Acta Anaesthesiol Scand, 2014. **58**(8): p. 914-22.

6. Australian, P.I.f.t., et al., *Effect of Stress Ulcer Prophylaxis With Proton Pump Inhibitors vs Histamine-2 Receptor Blockers on In-Hospital Mortality Among ICU Patients Receiving Invasive Mechanical Ventilation: The PEPTIC Randomized Clinical Trial.* JAMA, 2020. **323**(7): p. 616-626.

7. Kirkpatrick, A.W., et al., *Intra-abdominal hypertension and the abdominal compartment syndrome: updated consensus definitions and clinical practice guidelines from the World Society of the Abdominal Compartment Syndrome.* Intensive Care Med, 2013. **39**(7): p. 1190-206.

8. Patel, A., R.N. Kaleya, and R.J. Sammartano, *Pathophysiology of mesenteric ischemia.* Surg Clin North Am, 1992. **72**(1): p. 31-41.

9. O'Grady, J., *Acute liver failure.* J R Coll Physicians Lond, 1997. **31**(6): p. 603-7.

10. Banks, P.A., et al., *Classification of acute pancreatitis--2012: revision of the Atlanta classification and definitions by international consensus.* Gut, 2013. **62**(1): p. 102-11.

11. European Association for the Study of the, L., *EASL Clinical Practice Guidelines: management of cholestatic liver diseases.* J Hepatol, 2009. **51**(2): p. 237-67.

12. Khwaja, A., *KDIGO clinical practice guidelines for acute kidney injury.* Nephron Clin Pract, 2012. **120**(4): p. c179-84.

13. Horan, T.C., M. Andrus, and M.A. Dudeck, *CDC/NHSN surveillance definition of health care-associated infection and criteria for specific types of infections in the acute care setting.* Am J Infect Control, 2008. **36**(5): p. 309-32.

14. Singer, M., et al., *The Third International Consensus Definitions for Sepsis and Septic Shock (Sepsis-3).* JAMA, 2016. **315**(8): p. 801-10.

15. Robertson, G.L., *Diabetes insipidus: Differential diagnosis and management.* Best Pract Res Clin Endocrinol Metab, 2016. **30**(2): p. 205-18.

16. Yee, A.H., J.D. Burns, and E.F. Wijdicks, *Cerebral salt wasting: pathophysiology, diagnosis, and treatment.* Neurosurg Clin N Am, 2010. **21**(2): p. 339-52.

17. Spasovski, G., et al., *Clinical practice guideline on diagnosis and treatment of hyponatraemia.* Intensive Care Med, 2014. **40**(3): p. 320-31.

18. Subcommittee, A., T. American College of Surgeons' Committee on, and A.w.g. International, *Advanced trauma life support (ATLS(R)): the ninth edition.* J Trauma Acute Care Surg, 2013. **74**(5): p. 1363-6.
